# Laparoscopic ablation for liver malignancies: initial experience at a Scandinavian high volume HPB center

**DOI:** 10.1007/s00464-024-11125-x

**Published:** 2024-08-15

**Authors:** Jeanett Klubien, Lucas Alexander Knøfler, Peter Nørgaard Larsen, Susanne Dam Nielsen, Daisuke Fukumori, Jens Georg Hillingsø, Christoph Tschuor, Hans-Christian Pommergaard

**Affiliations:** 1 Department of Surgery and Transplantation, Rigshospitalet, Copenhagen University Hospital, Inge Lehmanns Vej 7, 2100 Copenhagen, Denmark; 2 Hepatic Malignancy Surgical Research Unit (HEPSURU), Department of Surgery and Transplantation, Rigshospitalet, Copenhagen University Hospital, Inge Lehmanns Vej 7, 2100 Copenhagen, Denmark; 3 Department of Infectious Diseases, Rigshospitalet, Copenhagen University Hospital, Esther Møllers Vej 6, 2100 Copenhagen, Denmark; 4 Institute for Clinical Medicine, Panum Institute, University of Copenhagen, Blegdamsvej 3B, 2200 Copenhagen, Denmark; 5 Department of Surgery and Transplantation, Rigshospitalet, University of Copenhagen, Inge Lehmanns Vej 7, 2100 Copenhagen Ø, Denmark

**Keywords:** Laparoscopic ablation, Microwave ablation, Liver malignancies, Liver tumors

## Abstract

**Background:**

Ablation is an effective, parenchymal-sparing treatment for primary liver cancer and liver metastases. The purpose of this study was to report our initial experience with laparoscopic microwave ablation regarding postoperative complications, rate of conversions to open procedure, and technical efficacy.

**Methods:**

This was a quality improvement project carried out at a tertiary care center in Denmark. Patients ≥ 18 years old with liver malignancies, not available for percutaneous ablation, and treated with ultrasound-guided laparoscopic ablation were included.

**Results:**

From March 2023 to December 2023, 39 patients were referred for laparoscopic ablation after a multidisciplinary team conference. Of these, two procedures were converted to open procedures due to adhesion and tumor progression. Three patients rejected the sharing of medical information, two procedures were canceled and in one case the strategy was changed perioperatively. Therefore, 32 procedures in 31 patients were available for analysis. Complete ablation was evaluated after 1 month and was achieved in 100% of the procedures. None of the patients died, and no complications were reported in 21 cases (65.6%). Most patients with complications had a grade 1 complication based on the Clavien–Dindo classification, which among others included abdominal and shoulder pain, atrial fibrillation, and subcutaneous hematoma. Two patients had a complication grade 2 (wound infection and decompensated cirrhosis) and one had a grade 4b (sepsis due to pneumonia and urinary tract infection). The median Comprehensive Complication Index was 12.2 (interquartile range 8.7–24.2). Furthermore, univariable logistic regression showed that ≥ 2 tumors treated were associated with a higher risk of complications (odds ratio 6.37, 95% confidence interval [1.20;33.85], *p*-value = 0.0297).

**Conclusion:**

Ultrasound-guided laparoscopic microwave ablation of liver malignancies is feasible and safe with little risk for complications, a high technical efficacy, and a low rate of conversions to open procedures.

**Supplementary Information:**

The online version contains supplementary material available at 10.1007/s00464-024-11125-x.

The liver is a common site for malignant abdominal disease including primary liver cancer and liver metastases [1, 2]. Microwave or radiofrequency ablation of liver tumors, a parenchymal-sparing procedure, can be notably beneficial for patients in need of repetitive treatment, particularly those with underlying liver disease such as cirrhosis, which carries a risk of complications including postoperative liver failure [3, 4]. Additionally, it may be preferred in frail patients who are unfit for major liver surgery [5–7]. Thus, ablation is the standard of care in patients with hepatocellular carcinoma for up to three tumors with a maximum diameter of 3 cm and sufficient liver function [8]. The procedure is repeatable in cases of incomplete treatment or recurrences, and it is a well-accepted strategy used as bridge to liver transplantation [9]. Ablation may be performed percutaneous or intraoperative by laparoscopy or laparotomy, and laparoscopy is currently the least used approach [10, 11]. In tumors not accessible for percutaneous ablation due to proximity to a hollow viscus or diaphragm, a laparoscopic approach may be more favorable compared with the open approach due to the less invasive nature of the procedure. This may potentially lead to fewer complications and shorter rehabilitation [12]. However, laparoscopic procedures as well as ultrasound-guided procedures are technically challenging and require practice and dedication to overcome the learning curve and achieve the needed level of competence [10, 13]. Thus, it therefore may be feared that the rate of complete ablation may be inferior to an open approach. However, the literature is limited on this topic.

The purpose of this study was to report our initial experiences with laparoscopic microwave ablation of liver tumors regarding the rate and severity of postoperative complications, conversion to open procedure as well as technical efficacy. Moreover, to provide perspectives and insights in the surgical management of patients with liver malignancies treated with ultrasound-guided laparoscopic microwave ablation.

## Materials and methods

### Study design and registrations

This was a prospective, observational single-center cohort study and a quality improvement project carried out at the tertiary care center, Copenhagen University Hospital, Rigshospitalet, in Denmark. All data were collected by one author (JK) and managed using Research Electronic Data Capture tools (REDCap) hosted at Rigshospitalet [14, 15]. REDCap is a secure, web-based software platform designed to support data capture for research studies, providing 1) an intuitive interface for validated data capture; 2) audit trails for tracking data manipulation and export procedures; 3) automated export procedures for seamless data downloads to common statistical packages; and 4) procedures for data integration and interoperability with external sources. After inclusion, baseline information was obtained from the patients’ medical records including patient and tumor characteristics, and perioperative and postoperative descriptions and information. Patients were included from March 2023 to December 2023 and the end of the follow-up period was ultimo January 2024. Each patient was followed until time of control imaging 4 weeks after the procedure as standard practice at this hospital.

The study and the retrieval of data were approved as a quality improvement project at Rigshospitalet (23071475), and according to Danish legislation, informed consent was not needed. The current study represents the initial experiences with laparoscopic ablation before inclusion in the randomized clinical trial comparing laparoscopic ablation and open ablation (the OPTIMAL study). It follows the same protocol except for contacting and interviewing patients pre- and postoperatively. The protocol for the OPTIMAL study is registered at Clinicaltrials.gov (NCT06304766). The reporting follows the Revised Standards for Quality Improvement Reporting Excellence (SQUIRE 2.0) for quality improvement in health care [16] and Strengthening the Reporting of Cohort Studies in Surgery (STROCSS) 2021 guideline for cohort studies in surgery [17].

### Participants and recruitment

Patients ≥ 18 years old with one or more liver malignancies were included. As an inclusion criterion, tumor(s) should not be accessible for percutaneous ablation due to proximity to critical structures, e.g., pericardium, diaphragm, or hollow viscera, or lack of visualization with percutaneous ultrasound with or without use of contrast. Furthermore, the decision to use ablation as the preferred treatment had to be made at a multidisciplinary team conference, where the factors considered included the above criteria, tumor localization, number and size of the tumors, and patient characteristics, including comorbidities and WHO performance status. The decision for patients with HCC was based on the BCLC staging systems stating ablation as a treatment option for patients with up to three tumors with a maximum diameter of 3 cm, and a Child–Pugh score of not more than B7 [8]. For patients with colorectal liver metastases, the criteria were poor performance status, comorbidities, and other contraindications for major surgery. All patients were identified using the hospital’s surgery management system by searching for laparoscopic ablation procedures. All cases were reviewed before inclusion.

### Intervention

The laparoscopic procedure was performed with the patient in general anesthesia. The Medtronic Emprint™ Ablation System was used. All procedures were guided by intraoperative ultrasound with the BK medical I13C3f (9076) Advanced Laparoscopic Transducer. This transducer has a laser feature which allows for guided navigation of the antenna through the abdominal wall and a needle guidance channel into the tumor. The laser-guided technique was mostly used for non-superficial tumors. This, however, was not feasible for the superficial tumors due to inability to adjust the angle of the track of the antenna into the tumor. Patients were positioned in supine position and with pneumoperitoneum through Palmer’s point, except for patients with tumors in segments 6 and 7. They were positioned with the right side up in a 45-degree angle and a pillow under the right side of thorax. The use of Veress needle or Visiport under the right curvature provided access to the abdominal cavity in these cases. Patients with tumors in segment 6/7 and other segment(s) were repositioned perioperatively for an optimal path. For the procedures, one 12 mm trocar and one to four 5 mm trocars were placed with local infiltration anesthesia. The abdomen is insufflated with carbon dioxide to a pressure of 12 mm Hg. First, a laparoscopic ultrasound of the liver was completed to locate the known tumor(s) and rule out any new tumors in the liver. A biopsy for pathological analysis was performed before ablation when the radiological or clinical diagnosis was uncertain. After identification of tumors, in some cases using contrast enhanced visualization with SonoVue, the microwave antenna was placed through the skin and in the tumor under real-time ultrasound-guidance. A 1-min preburn with 45W was done initially. Thereafter, for each tumor, watts (W) and minutes were adjusted with the freeware app Emprint™ Ablation System to get a rim of 1 cm ablated margin of normal liver parenchyma. Track ablation was done in the ending of all procedures. In cases where perioperative ultrasound did not show complete ablation of the tumor, overlapping retreatment were performed. If necessary, the liver and/or adjacent organs were mobilized, further, saline and interpositioned gauzes were used to protect adjacent organs from thermal injury. In the few cases where tumors were located closely to the gallbladder, a cholecystectomy was performed. All cases were done by author CT and/or HCP. CT underwent laparoscopic training during several minimally invasive HPB fellowships in the United Kingdom, Italy, and the United States. CT learned the procedure during his robotic HPB fellowship at Carolinas Medical Center in Charlotte. HCP has previous general laparoscopic and liver ultrasound experience and was trained for the procedure by CT.

### Postoperative plan and follow-up

Patients were admitted on the day of the procedure and followed a standardized pre- and postoperatively plan. Routine blood tests were measured before the procedure and on the first postoperative day. In relevant cases, patients with cirrhosis were postoperatively treated with Albumin infusion and Spironolactone as fluid therapy and to prevent renal decompensation and ascites formation, respectively. Furthermore, treatment with Selective Non-Steroidal Anti-Inflammatory Drugs (Celecoxib) and Paracetamol were prescribed for the first 3 days postoperative. If needed, Morphine 5 mg was added for 3 days. After discharge, a nurse contacted the patients by telephone twice within 14 days with questions regarding: pain, surgical wound, mobilization, nutrition, and gastrointestinal function. The technical efficacy was evaluated with control imaging 4 weeks later.

### Definitions of endpoints

The endpoints technical efficacy and complications were defined as follows: technical efficacy (complete ablation response) was defined as non-enhancement of the ablated area on a multi-phase liver CT four weeks after the procedure [18]. The CT was reviewed by radiologists with dedicated experience in hepatobiliary imaging. Complications were recorded from the day of the procedure and until 30 days after. Complications was categorized using the Clavien–Dindo classification [19, 20]. With this classification, the severity of the complication is graded based on the type of therapy needed to correct it. The scale consists of the following grades 1, 2, 3a, 3b, 4a, 4b, and 5. Furthermore, we used the Comprehensive Complication Index (CCI) [21, 22], which is based on the Clavien–Dindo classification, and provides a sum of all complications with a score from 0 to 100.

### Statistical analysis

The sample size was determined by the number of eligible procedures in the study period prior to initiating an RCT, and therefore, no power calculation was done prior to inclusion. For the continuous data, proportions reported with frequencies were used and for the continuous data, median with range or interquartile range (IQR) were used. Descriptive statistics were used to report the endpoints of interest including reasons for conversion from laparoscopic to open procedure, complications, and technical efficacy. Moreover, univariable logistic regression was used to examine if available patient- and tumor-related factors were associated with complications (none vs. grade ≥ 1) at first treatment. Results were reported with odds ratio and corresponding 95% confidence interval. A paired t-test was performed to examine for a difference in pre- and postoperative available blood tests (bilirubin, albumin, INR, creatinine, and sodium). All statistical analyses were completed with R, version 4.2.2.

## Results

### Participants

In total, 39 patients were planned for laparoscopic ablation after a multidisciplinary team conference in the study period. Of these, two procedures were converted to open hepatic resection perioperatively. In the first case, due to progression to a tumor size and a difficult localization of the tumor, and in the other due to massive adhesion after previous abdominal surgery (5.1%). The strategy was changed perioperatively in one case since the metastases were not identified as tumor tissue macroscopically or by ultrasound. This led to a laparoscopic local hepatic resection for a minor subcapsular lesion (4 mm) for histological confirmation (2.6%). Two procedures were canceled; one due to progression noted on preoperative CT scan and the other due to decompensated liver disease with ascites in a patient with hepatocellular carcinoma (HCC) (pre-operative Child–Pugh B7, and Child–Pugh B9 at the time of surgery due to decompensation) (5.1%). Lastly, three patients had disclosed in their medical records that they rejected the sharing of information for research purposes including quality improvement (7.7%).

Ultimately, of the initial 39 patients, we included 31 patients that underwent ultrasound-guided laparoscopic microwave ablation. One of the 31 patients with HCC was treated twice for two separate primary tumors. As a result, a total of 32 procedures in 31 patients were available for analysis. Baseline characteristics of the included patients at time of the first procedure are shown in Table 1. Male sex was prominent (84%) as were HCC as primary tumor (58%). Of 18 patients with HCC, 17 had liver cirrhosis and the most common etiology was alcohol-related and chronic infection with hepatitis B or C. The Child–Pugh score was unknown in one case, 15 patients had a Child–Pugh score A, and one had a Child–Pugh score B7.

**Table 1:** Baseline patient and tumor characteristics at time of first procedure

	*n* = 31
Male sex, *n* (%)	26 (84)
Age in years, median (IQR)	71.3 (64.4–75.1)
Body mass index, median (IQR)	26.1 (23.6–30.0)
Current smoker, *n* (%)	10 (32.3)
Alcohol consumption, *n* (%)	
None	10 (32.3)
Less or equal to recommended 10 units a week	14 (45.2)
More than recommend 10 units a week	7 (22.6)
WHO performance status, *n* (%)	
0	18 (58.1)
1	9 (29.0)
2	4 (12.9)
Primary cancer, *n*	
Hepatocellular carcinoma	18
Liver metastases	
Colorectal cancer	9
Anal cancer	2
Neuroendocrine tumor	2
Number of tumors, median (range)	1 (1–3)
Diameter of largest tumor, median (range)	1.7 (0.7–3.0)
Liver segments involved (primary tumor)	
Hepatocellular carcinoma	2, 3, 4a, 4b, 5, 6, 7, 8
Colorectal cancer	3, 4b, 5, 6, 7, 7/8
Neuroendocrine tumors	7, 8
Anal cancer	2, 7
Prior chemotherapy, *n* (%)	3 (9.7)
Prior ablation, *n* (%)	5 (16.1)
Prior abdominal surgery, *n* (%)	15 (48.4)

*IQR* interquartile range

### Procedure

The indication for laparoscopic microwave ablation and/or why a percutaneous approach was not possible were (with some overlapping between the following): tumor not visualized and/or not accessible percutaneously (*n* = 14), size of tumor too large for percutaneous ablation (*n* = 2), and tumor location near gallbladder, diaphragm, colon, or stomach (*n* = 20).

The median duration of the procedures from incision-to-close was 80 min (IQR 56–101). Power for the microwave ablation ranged from 75 to 100W and number of minutes per tumor depended on size and ranged from 1 to 8 min. In three cases, the position of the antenna was changed with subsequent ablation due to suspicion of ineffective treatment perioperatively. The median length of hospital stay was 1 day with a range from 0 to 5 days. None of the patients underwent further surgery in the 30-day period after the procedure.

### Technical efficacy

All 32 procedures achieved complete ablation with no viable tumor tissue left at 4 weeks post-intervention, resulting in a success rate of 100%.

### Complications

To report complications, all included patients were followed for 30 days, except for one patient only followed for complications until day 14 as the patient has residence outside of Denmark. No patients died in the first 30 days postoperatively. Of all the 32 procedures, no complications were reported in 21 cases (65.6%). Of the reported complications, the majority had a grade 1 complication. A description of complications is reported in Table 2. Three patients had a complication graded ≥ 2. One of these patients was readmitted with sepsis (grade 4b complication), but fully recovered after discharged.

**Table 2:** Descriptive report on complications graded by the Clavien–Dindo classification

Clavien–Dindo classification	Description	No. of patients
1	Abdominal pain (defined by additional need of analgesics)	2
1	Hypotension and slightly affected renal function	1
1	Subcutaneous hematoma and affected liver function without requirement of treatment	1
1	Atrial fibrillation and bradycardia during the procedure	1
1	Shoulder pain	1
1	Abdominal pain and erythema caused by allergic reaction to bandage	1
1	A small lesion of liver capsule with CO2 in systemic circulation without need for additional treatment	1
2	Surgical wound infection	1
2	Decompensated cirrhosis with postoperative ascites	1
4b	Sepsis due to pneumonia and urinary tract infection, complicated by pulmonary edema and acute kidney injury	1

The CCI was calculated based on the reported grades from the Clavien–Dindo classification, and the median score was 12.2 (IQR 8.7–24.2). This corresponds to the majority experiencing one or two grade 1 complication. Furthermore, the unadjusted logistic regression showed that more than one tumor treated was associated with a higher risk of complications (odds ratio 6.37, 95% confidence interval [1.20; 33.85], *p*-value = 0.0297). None of the other factors examined were significantly associated with complications (Table 3).

**Table 3:** Univariable logistic regression for complications (Clavien–Dindo classification ≥ 1) for patients treated with laparoscopic ablation for liver malignancies

	OR (95% CI)	*p*-value
Age	1.04 (0.96;1.14)	0.329
Sex		
Female	Ref	
Male	4.07 (0.56;29.73)	0.166
Current alcohol consumption		
0–10 units a week	Ref	
> 10 units a week	0.80 (0.13;5.07)	0.813
Current smoker		
No	Ref	
Yes	0.86 (0.17;4.3)	0.853
Body mass index	1.08 (0.92–1.28)	0.346
Number of tumors		
1	Ref	
≥ 2	6.37 (1.20;33.85)	0.0297
Diameter of largest tumor	1.33 (0.42;4.26)	0.629
Performance status		
0	Ref	
≥ 1	1.62 (0.36–7.43)	0.532
Previous surgery	1.33 (0.29;6.04)	0.709

*OR* odds ratio, *CI* confidence interval, *Ref.* reference

There was no significant change in the pre- and postoperative values for bilirubin, sodium, creatine, or INR. Unfortunately, albumin was not part of the routine blood tests.

## Discussion

In this study of patients treated with laparoscopic microwave ablation for liver malignancies, two cases were converted to open resection. In one case it was due to extensive adhesions after previous abdominal surgery, and in the other, it was due to progression and a difficult localization of the tumor. In the 32 laparoscopic microwave ablations performed, complete ablation was achieved in 100% of patients assessed 4 weeks after surgery. No deaths were observed, and only minor complications were reported. The most frequently reported complication was one or more grade 1 complications represented in a low median CCI score of 12.2.

Short- and long-term outcomes after laparoscopic ablation for liver malignancies with different primary tumors are increasingly being reported with good results [23]. In this study, we reported our initial experiences and short-term outcomes. We found a very high technical efficacy. Other recent studies also reported high rates of complete ablation response after laparoscopic ablation in 92–100% of the procedures [13, 24–28]. One study found risk factors for incomplete ablation to be the invisibility of the tumor on laparoscopy and peri-hepatic vein location [29]. The complication rate and severity of complications after laparoscopic ablation are slightly more difficult to compare since most studies defined complications as minor or major and did not use a standardized classification system as, e.g., the Clavien–Dindo classification. However, other studies with no procedure-related mortality reported major complication rates of 5.9–14.3% [13, 24, 25, 27]. A large study of laparoscopic radiofrequency ablation of liver malignancies reported a 30-day mortality rate of 0.4% and a morbidity rate of 3.8% [30]. When comparing to open ablation, a study from the same institute found a complication rate (grade > 2 by the Clavien–Dindo) of 45% for patients with colorectal liver metastases, but this was in a small cohort [31]. Another study reporting complications by Clavien–Dindo classification after open ablation for liver metastases found an overall rate of complications of 39.5% and for grade 3 or higher of 18.4% [32]. In the current study, patients with cirrhosis were included. These patients are potentially at a higher risk for complications after surgical interventions due to the underlying liver disease [3]. However, underlying liver disease did not increase the rate of complications in our study. The technical efficacy in the current study is comparable with results after open ablation of 96% found in a nationwide study on ablation for hepatocellular carcinoma [11]. The study reported a complication rate (Clavien–Dindo classification grade ≥ 2) of 25.4% after open ablation. Three patients in the current study had a grade ≥ 2 complication (9.4%), two of these patients had more than one tumor treated.

There are several strengths of this study. Firstly, there was practically complete data and follow-up except for just one patient who was only followed until day 14 for complications, but the control imaging of this patient was performed 4 weeks after the procedure. The data for this study were documented prospectively as part of standard clinical practice. Secondly, the protocol was preregistered at Clinicaltrials.gov. Thirdly, the SQUIRE 2.0 and STROCSS 2021 guidelines were used for reporting to ensure transparency. The limitations are mainly due to the design as a single-center study with a short study period and a small, heterogeneous cohort of included patients. The purpose of this article was, however, to report the technical management of patients with liver malignancies treated with laparoscopic ablation and to ensure the feasibility and safety of the procedure prior to including patients in the RCT (OPTIMAL study). This study demonstrates that laparoscopic ablation is well-tolerated with low morbidity despite the cohort’s heterogeneity for primary cancer, age, comorbidities, and previous surgery.

Outcomes after ablation are highly dependent on the interventional radiologist’s or surgeon’s ability to accurate locating tumors and correctly place the antenna, otherwise, it may lead to incomplete ablation. This may be crucial for the prognosis of the patient after ablation [33]. Tumors located deeper in the parenchyma may particularly be an issue. With the development of new techniques, laparoscopic ablation may be easier to perform. In this study, the BK Laparoscopic Transducer, equipped with a laser feature and a guidance channel, assists the surgeon in accurately placing the antenna within the tumor. This contrasts with conventional laparoscopic ablation, which relies only on the surgeon’s assessment. These techniques, however, still operate in a two-dimensional space and the accuracy of antenna placement are highly dependent on the surgeon’s expertise and ability to localize the antenna in relative to the tumor and other structures. In relation to this, the Emprint™ SX system uses a three-dimensional tracking for intraoperative assistance for antenna guidance, but this has yet to be marketed in Europe [34]. This demonstrates ongoing advancements in the techniques used for laparoscopic ablation, suggesting that the laparoscopic approach may be even easier to perform with concomitantly good treatment outcomes. Furthermore, an advantage of the laparoscopic approach is that the surgeon has the possibility for intraoperative restaging and thereby detecting new tumors not identified by preoperative imaging in a minimally invasive fashion. It also allows for faster handling of any intraoperative complications that can arise. Appropriate patient selection for the optimal approach may help minimize the risk of complications including conversions, while maintaining the high technical efficacy of the treatment. The OPTIMAL study comparing open and laparoscopic ablation will investigate the complication rate, technical efficacy, quality of life and recovery, and cost-effectiveness of the two approaches. This may contribute to understanding and thereby including a larger group of patients benefitting from a minimal invasive approach.

In conclusion, ultrasound-guided laparoscopic microwave ablation of liver malignancies is feasible and safe with little risk for complications, a high technical efficacy, and a low rate of conversions to open procedure.

## Supplementary Information

Below is the link to the electronic supplementary material.

Supplementary file1 (MOV 176831 KB)
